# Jarid2 is essential for the maintenance of tumor initiating cells in bladder cancer

**DOI:** 10.18632/oncotarget.15522

**Published:** 2017-02-20

**Authors:** Xin-Xing Zhu, Ya-Wei Yan, Chun-Zhi Ai, Shan Jiang, Shan-Shan Xu, Min Niu, Xiang-Zhen Wang, Gen-Shen Zhong, Xi-Feng Lu, Yu Xue, Shaoqi Tian, Guangyao Li, Shaojun Tang, Yi-Zhou Jiang

**Affiliations:** ^1^ Institute for Advanced Study, Shenzhen University, Shenzhen, Guangdong, China; ^2^ Key Laboratory of Optoelectronic Devices and Systems of Ministry of Education and Guangdong, College of Optoelectronic Engineering, Shenzhen University, Shenzhen, Guangdong, China; ^3^ Department of Statics, University of Wisconsin-Madison, Madison, WI, USA; ^4^ Maternal and Child Health Hospital of Nanshan District, Shenzhen, Guangdong, China; ^5^ The First Affiliated Hospital of Xinxiang Medical University, Weihui, Henan, China; ^6^ Department of Physiology, Center for Diabetes, Obesity and Metabolism, Shenzhen University, Shenzhen, Guangdong, China; ^7^ Minnan Normal University, Zhangzhou, Fujian, China; ^8^ The Affiliated Hospital of Qingdao University, Qingdao, Shandong, China; ^9^ Department of Health Outcomes and Policy, College of Medicine, University of Florida, Gainesville, FL, USA; ^10^ Innovation Center for Biomedical Informatics, Georgetown University Medical Center, Washington, DC, USA

**Keywords:** Jarid2, bladder tumors, tumor-initiating cells, p16, histone modification

## Abstract

Bladder cancer is the most common urologic malignancy in China, with an increase of the incidence and mortality rates over past decades. Recent studies suggest that bladder tumors are maintained by a rare fraction of cells with stem cell proprieties. Targeting these bladder tumor initiating cell (TICs) population can overcome the drug-resistance of bladder cancer. However, the molecular and genetic mechanisms regulating TICs in bladder cancer remain poorly defined. Jarid2 is implicated in signaling pathways regulating cancer cell epithelial-mesenchymal transition, and stem cell maintenance. The goal of our study was to examine whether Jarid2 plays a role in the regulation of TICs in bladder cancer. We found that knockdown of Jarid2 was able to inhibit the invasive ability and sphere-forming capacity in bladder cancer cells. Moreover, knockdown of Jarid2 reduced the proportion of TICs and impaired the tumorigenicity of bladder cancer TICs *in vivo*. Conversely, ectopic overexpression of Jarid2 promoted the invasive ability and sphere-forming capacity in bladder cancer cells. Mechanistically, reduced Jarid2 expression led to the upregulation of p16 and H3K27me3 level at p16 promoter region. Collectively, we provided evidence that Jarid2 via modulation of p16 is a putative novel therapeutic target for treating malignant bladder cancer.

## INTRODUCTION

Bladder cancer is one of the most prevalent cancers among males, and its high mortality mirrors problems with aggressiveness and drug-resistance [1]. In a variety of solid tumors, tumor-initiating cells (TICs) have been implicated in therapeutic resistance and relapse after initial therapy [2]. Hence, better understanding of how TICs differ from non-TIC cancer cells and how TICs contribute to relapse and resistance will support the development of effective therapeutics against bladder cancer. Several markers, such as ALDH activity, expression of Bmi1 and Sox2, are commonly used as markers in identification and characterization of TICs in bladder cancer [3–5]. However, the molecular features of TICs in bladder cancer remain poorly defined.

Jarid2 belongs to AT-rich interaction domain containing (ARID) gene family, which can modify chromatin structure through DNA binding and regulate targeted gene transcription. ARID gene family plays important roles in cancer-related signaling pathways and are highly mutated or differentially expressed in cancer cells [6]. Jarid2 also contains a Jumonji domain but lacks histone demethylase activity. Jarid2 can interact with Polycomb repressive complex-2 (PRC2) to regulate the maintenance of pluripotency and differentiation of embryonic stem cells [7–9], suggesting a function of Jarid2 in stem cell biology. Study has also shown that Jarid2 is required TGF-β-induced epithelial-mesenchymal transition (EMT) through repression of CDH1 and miR-200 family genes in lung and colon cancers [10]. In addition, JARID2 positively mediates EMT of hepatocellular carcinoma via PTEN/AKT signaling [11], indicating a role of Jarid2 in the metastatic property of cancer cells. However, the roles of Jarid2 in bladder cancer cells have remained elusive.

In current study, we have analyzed the expression of ARID gene family in TICs of bladder cancer cell lines. We have characterized the role of Jarid2 over cell invasion, sphere-forming ability of bladder cancer and tumorigenicity of TICs. Furthermore, we have studied the effect of Jarid2 on the expression of p16, a known PRC1/2 target gene.

## RESULTS

### Jarid2 is enriched in TICs of bladder cancer

**Figure 1:**
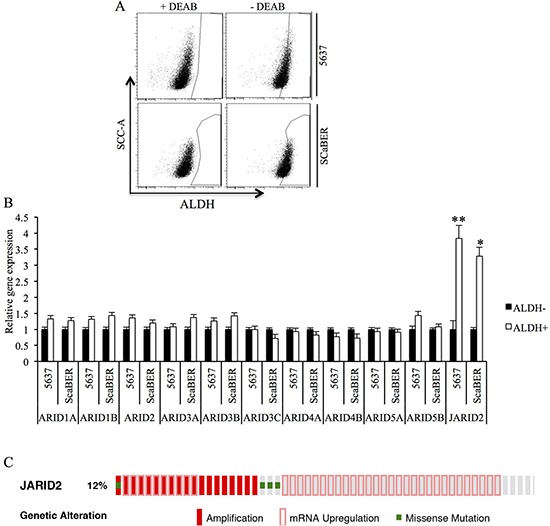
Jarid2 is enriched in TICs of bladder cancer cell lines **(A)** Representative Aldeflour assay result of 5637 and SCaBER cancer cells showed 7.8% and 15.8% ALDH+ subpopulation, respectively. **(B)** Expressions of ARID family were measured by qRT-PCR in ALDH^high^ and ALDH^low^ populations isolated from 5637 and SCaBER cell lines. **(C)** Jarid2 alteration in 413 bladder cancer patients based on cBioportal website. **P < 0.01; *P < 0.05 is based on the Student *t* test. All results are from biological triplicates. Error bars, standard deviation (*n* = 3).

We first isolated TICs based on ALDH activity in two bladder cancer cell lines. Flow cytometry demonstrated heterogeneous ALDH activity among different bladder cancer cell lines with 7.8% ALDH^high^ cells in 5637 cell line relative to DEAB control samples, whereas 15.8% ALDH^high^ cells were isolated from SCaBER cell line (Figure 1A). We next evaluated ALDH^high^ and ALDH^low^ cell populations for expression of ARID gene family. For the expression other ARID gene family members, we did not observe any significant changes (Figure 1B). However, JARID2 expression was shown clearly increased in ALDH^high^ cells from both cell lines (Figure 1C). Next, The Cancer Genome Atlas (TCGA) data were accessed and analyzed via the eBioPortal for Cancer Genomics (http://www.cbioportal.org) for the alteration of Jarid2 expression in 412 bladder cancer patients. As shown in Figure 1C, the expression of Jarid2 is altered in 51 out of 412 cases (12.3%). Together, our results suggest that Jarid2 might play a role in the regulation of TICs in bladder cancer.

### Jarid2 expression was positively associated with bladder cancer cell invasion and sphere-forming ability

**Figure 2:**
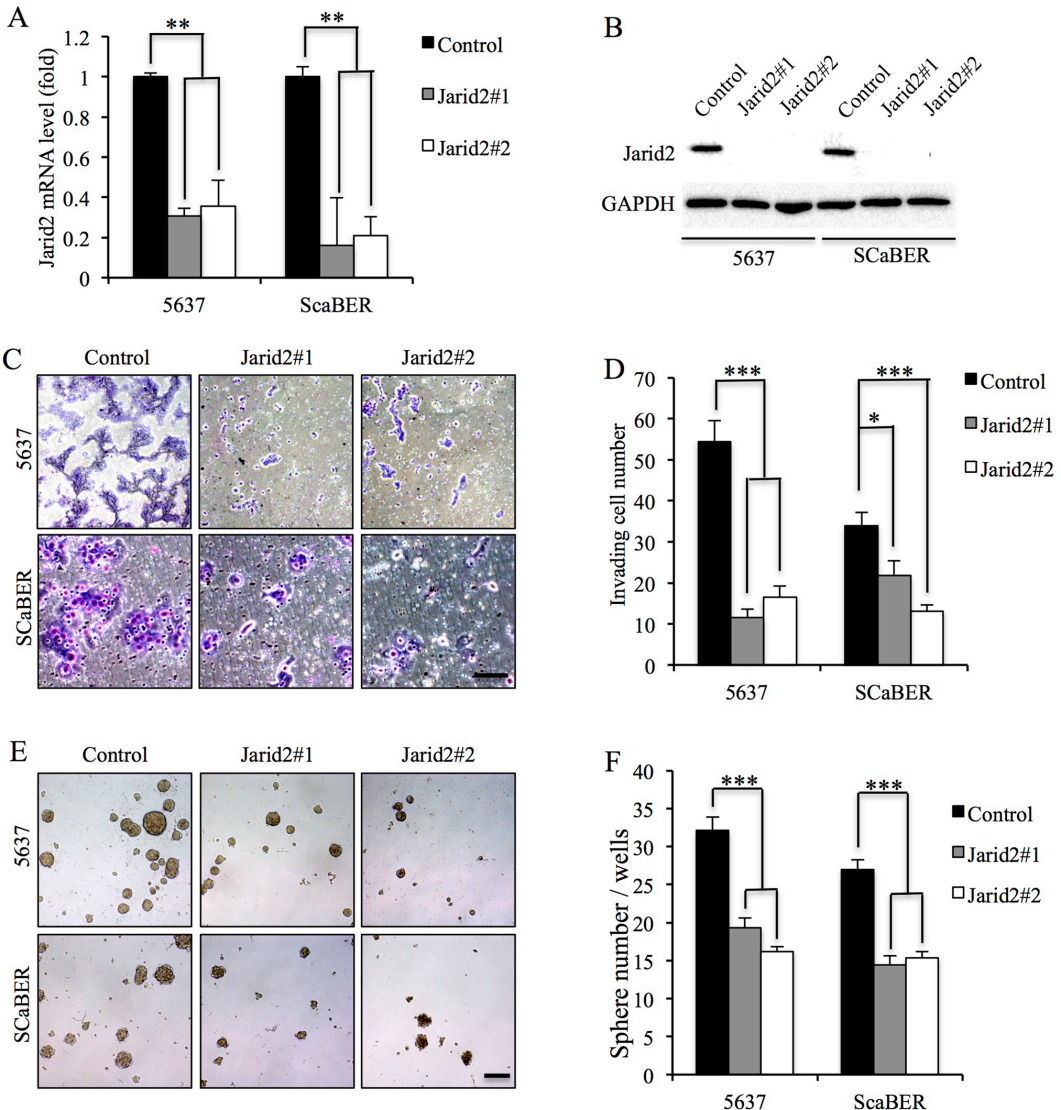
Knockdown of Jarid2 led to decrease invasive capacities and sphere-forming ability in bladder cancer cell lines **(A)** Jarid2 knockdown efficiency was measured by qRT-PCR in 5637 and SCaBER cell lines. **(B)** Jarid2 knockdown efficiency was measured by Western blotting in 5637 and SCaBER cell lines. **(C, D)** Control and Jarid2-silencing 5637 and SCaBER cells were subjected to Matrigel invasion assays, quantification of invaded cells through Matrigel of each cell line are shown as proportions of their siRNA controls. Scale bar, 50 μm. **(E, F)** Representative microscopy images of tumor sphere formation and quantification of tumor spheres formed in control and Jarid2-silencing 5637 and SCaBER cells. Scale bar, 200 μm. ***P < 0.001; **P < 0.01; *P < 0.05 is based on the Student *t* test. All results are from biological triplicates. Error bars, standard deviation (*n* = 3).

**Figure 3:**
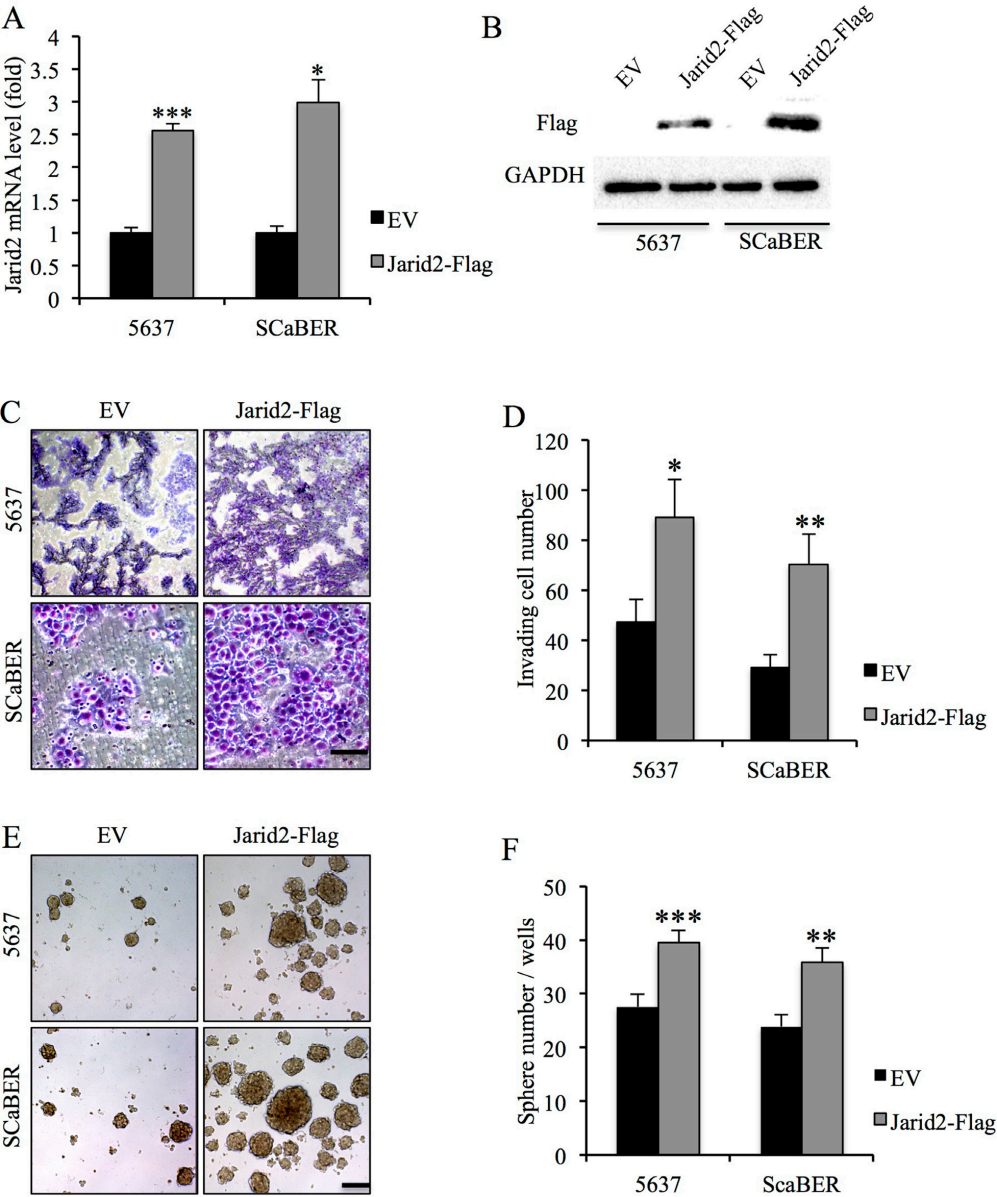
Ectopic over-expression of Jarid2 promotes invasive capacities and sphere-forming ability of bladder cancer cells *in vitro* **(A)** qRT-PCR assessment of elevated expression of Jarid2 mRNA in bladder cancer cell lines stably transfected with Jarid2-Flag in comparison with cells transfected with empty vector alone. **(B)** Western blotting assessment of elevated expression of Jarid2 protein in bladder cancer cell lines stably transfected with Jarid2-Flag in comparison with cells transfected with empty vector alone. **(C, D)** Jarid2-over-expressing bladder cancer cells and their control vector cells were subjected to Matrigel invasion assays, quantification of invaded cells through Matrigel of each cell line are shown as proportions of their vector controls. Scale bar, 50 μm. **(E, F)** Representative microscopy images of tumor sphere formation and quantification of tumor spheres formed in control and Jarid2-overexpressing 5637 and SCaBER cells. Scale bar, 200 μm. ***P < 0.001; **P < 0.01; *P < 0.05 is based on the Student *t* test. All results are from biological triplicates. Error bars, standard deviation (*n* = 3).

To further investigate the function of Jarid2, we examined the cell proliferation, invasion and sphere-forming capacity in 5637 and SCaBER cells with down-regulated or over-expressed Jarid2 (Figure 2A, 2B). We detected no significant difference in cell proliferation in control and Jarid2 knocked down in both cell lines (Supplementary Figure 1). However, invasion and sphere-forming experiments showed that down-regulation of Jarid2 significantly inhibited cell invasion and sphere-forming ability in both cell lines (Figure 2C–2F). Conversely, when Jarid2-Flag plasmid was transfected into the 5637 and SCaBER cell lines, invasion and sphere formation data showed the opposite results (Figure 3A–3F). In summary, our data indicated that Jarid2 expression was positively associated with bladder cancer cell invasion and sphere-forming ability *in vitro*.

### Jarid2 knockdown reduces ALDH activity and tumorigenicity of bladder cancer cell

**Figure 4:**
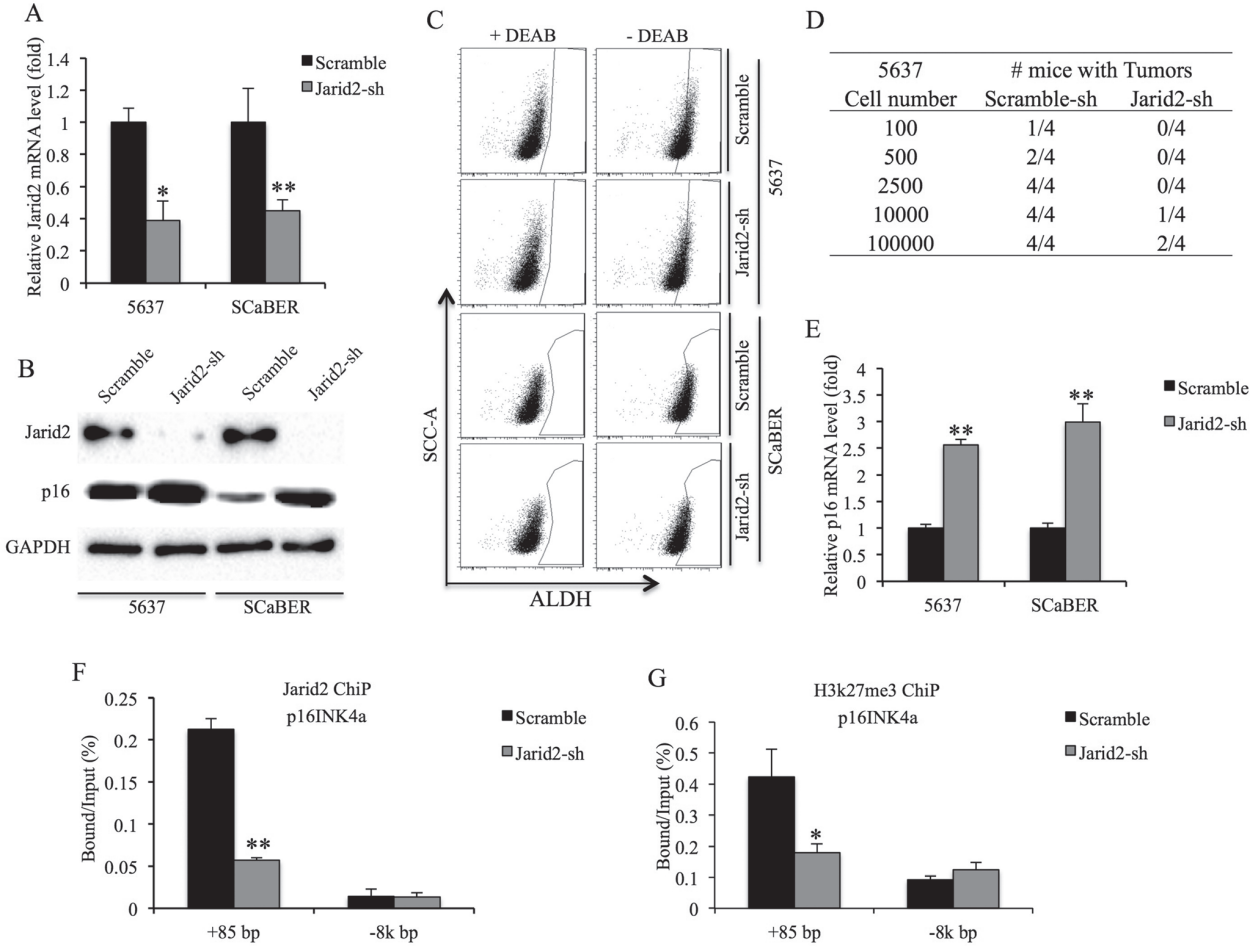
Knockdown of Jarid2 impairs bladder cancer TICs tumorigenicity *in vivo* **(A)** Jarid2 knockdown efficiency was measured by qRT-PCR in 5637 and SCaBER cell lines. **(B)** Jarid2 knockdown efficiency and p16 protein expression were measured by Western blotting in 5637 and SCaBER cell lines. **(C)** ALDH activity is suppressed in Jarid2-shRNA infected cells, compared to control cells. **(D)** Summary of results of limiting dilution experiments to assess frequency of tumor formation generating by ALDH^high^ TICs from control and Jarid2-sh 5637 cells. **(E)** Expression of p16 mRNA was measured by qRT-PCR in Jarid2-shRNA infected cells, compared to control cells. **(F)** ChIP was used to detect protein binding to p16 promoter using antibodies to Jarid2 or IgG control. ChIP DNA was analyzed by real-time PCR at the promoter region or non-target downstream of p16 gene in control or Jarid2-shRNA infected 5637 cells. **(G)** ChIP was used to detect protein binding to p16 promoter using antibodies to H3K27me3 or IgG control. ChIP DNA was analyzed by real-time PCR at the promoter region or non-target downstream of p16 gene in control or Jarid2-shRNA infected 5637 cells. ***P < 0.001; **P < 0.01; *P < 0.05 is based on the Student *t* test. All results are from biological triplicates. Error bars, standard deviation (*n* = 3).

To examine the function of Jarid2 in the ALDH activity and tumorigenicity of bladder cancer cells, we obtained stable bladder cell lines with lentivirus-mediated shRNA knockdown of Jarid2 compared with control cells expressing shGFP (Figure 4A, 4B). The control 5637 and SCaBER cells contained 7.5% and 15.3% ALDH^high^ cells, respectively, which were comparable with their uninfected parental cells (Figure 1A and Figure 3C). In contrast, we only detected 2.4% and 5.8% ALDH^high^ cells in Jarid2-sh 5637 and SCaBER cells, respectively (Figure 3C), suggesting that the Jarid2 is necessary for ALDH activity in bladder cancer cells *in vitro*. To examine whether Jarid2 is critical for the tumorigenicity of bladder cancer TICs *in vivo*, we performed limiting dilutions (100, 500, 2500, 1 × 10^4^ and 1 × 10^5^) of ALDH^high^ cell sorted from stable Jarid2-sh 5637 and SCaBER cells. As shown in Figure 4D, fewer tumors formed in Jarid2-sh cells compared to control cells when 10^5^ or 10^4^ cells were implanted. In contrast, no tumors formed in the 5637 cells with silencing of Jarid2-sh when 100, 500, 2500 cells were injected (0/4), while tumors still formed in control injections (Figure 4D). These results combined, support the sphere-forming data, suggesting that Jarid2 is required for the maintenance of the stem cell population in bladder cancer.

### Jarid2 inhibits the expression of p16 in TICs

p16^INK4a^, a known tumor suppressor which is epigenetically repressed by PRC2 and PRC1, can induce senescence and depletion of stem cells [12, 13]. Loss of Jarid2 in embryonic stem cells dramatically inhibits binding of the PRC2 to their target genes [14]. Interestingly, our results showed the level of p16^INK4a^ transcript and protein was up-regulated in both Jarid2-sh bladder cancer cell lines (Figure 4B and 4E). ChIP assay results from 5637 cells showed Jarid2-sh cells had reduced levels of Jarid2 and H3K27me3 in the promoter region of p16^INK4a^ (Figure 4F, 4G). In summary, Jarid2 can localize to the promoter region of p16^INK4a^ and is required for H3K27me3 modification of this region.

## DISCUSSION

Bladder cancer is the fourth most common malignancy in men. The 5-year survival rate of bladder cancer patients ranges between 40% and 60% and has not been improved in the last decade. It is becoming increasingly clear that TICs in tumors cannot be eradicated by traditional chemotherapy, which causes the tumor recurrence and poor survival [15]. In this study, we found that Jarid2 was enriched in the TICs of two bladder cancer cell lines. Jarid2 gene encodes a protein that contains a Jumonji- and AT-rich interaction domain (ARID)-domain. Jarid2 can be associated with PRC1/2 and acts as a transcriptional repressor in embryonic stem cells [14, 16]. Jarid2 facilitates the recruitment of the PRC1/2 complex to target genes and plays a critical role in regulating gene expression during embryonic development [7, 17].

Functionally, we found Jarid2 is required for the invasive ability and sphere-forming capacity in bladder cancer cells. Silencing of Jarid2 also decreased the percentage of TICs and inhibited the tumorigenicity of bladder cancer TICs. Complementarily, forced expression of Jarid2 promoted the invasive ability and sphere-forming capacity in bladder cancer cells. In lung and colon cancer, Jarid2 is involved in EMT process induced by TGF-β through EZH2-mediated transcriptional repression of CDH1 and microRNA-200 family genes [10]. Moreover, JARID2 can regulate cell migration, invasion, proliferation and metastasis of hepatocellular carcinoma by repressing expression of tumor suppressor gene PTEN via increasing H3K27me3 level at PTEN promoter region [11]. Consistently, our data showed that reduced Jarid2 expression led to the upregulation of p16 and H3K27me3 level at p16^INK4a^ promoter region. Previous study showed that loss of tumor suppressor gene p16^INK4a^ confers the stem-cell-like property and therapeutic resistance in human breast cancer [18].

## MATERIALS AND METHODS

### Cell culture and sphere culture

Bladder cancer cell lines 5637 and SCaBER purchased from Shanghai Institute of Biochemistry and Cell Biology (Shanghai, China) were cultured in Dulbecco's modified Eagle's medium (DMEM) containing 10% fetal bovine serum (FBS) and antibiotics at 37°C in 5% CO_2_ atmosphere. For sphere forming assay, 2,000 cells were plated into Ultra-Low Attachment 24-well culture plates (Corning) and cultured in a serum-free DMEM/F12 supplemented with B-27 Supplement, 20 ng/mL basic fibroblast growth factor, 10 ng/mL epidermal growth factor and antibiotics. Fresh medium was added every two or three days, and spheres were cultured for 20 day.

### RNA interference and lentivirus transfection

siRNA-mediated gene silencing studies were performed as previously described [19]. The sequences of siRNA against Jarid2 are: CCACACAAUCUCAGGGAAA, AGGAAGAGGAGGAGGACAA. Jarid2 shRNA lentiviral plasmid was purchased from Sigma (SHCLND-NM_004973). To obtain stable cell lines, 5637 and SCaBER cells transfection with lentivirus were selected with medium containing 2 ug/ml puromycin for 7 days.

### Cell proliferation and invasion assays

For proliferation assay, 2.5 × 10^3^ 5637 or SCaBER cells were seeded in 96-well plates. Cell proliferation was measured every day for 6 days by an MTT [3-(4,5-dimethylthiazol-2-yl)-2,5-diphenyltetrazolium bromide] assay (Thermo Fisher Scientific) using the manufacturer's guidance. For invasion assay, 200 μl of 5 × 10^5^ bladder cancer cells were seeded in triplicate 24-well transwell chambers coated with matrigel (EMD Millipore). The cells were plated in medium without serum. The lower chamber was filled with 600 μl conditioned medium (DMEM medium containing 1% FBS for 24 h) as chemoattractant. After 24 h incubation, the cells invaded to the lower surface of the filter were fixed with methanol, stained with hematoxylin and quantified by six random fields of view.

### RNA extraction and real-time RT-PCR

Total RNA was extracted from cells with TRIzol reagent (Invitrogen Life Technologies, China) following the manufacturer's instructions. The real-time quantitative PCR reaction was performed as previously described [20, 21]. The relative expression of target transcripts was normalized against that of β-actin. The primers for these transcripts are listed in Supplementary Materials.

### Immunoblotting

Immunoblotting was performed as previously described [22]. Anti-Flag (Cat. F7425) was purchased from Sigma. Anti-GAPDH (Cat. sc-47724) was purchased from Santa Cruz Biotech. Anti-Jarid2 (Cat. ab48137) and anti-p16 (Cat. ab201980) were purchased from Abcam.

### Aldefluor assay and FACS

The Aldefluor assay (Stem Cell Technologies) was used to profile and sort cells based on ALDH activity as previously described [19]. ALDH^high^ and ALDH^low^ cells were sorted using BD Aria II (BD Biosciences) cell sorters. Flow cytometric profiling was carried out on a FACScan flow cytometer (BD Biosciences) and analyzed by FlowJo software (Treestar).

### Chromatin immunoprecipitation assay

ChIP experiments were performed as previously described [19]. The crosslinked chromatins were immunopreciptated with anti-H3K27me3 (Cat. ab6002) and anti-Jarid2 (Cat. ab48137) antibodies from Abcam. The enrichment of the specific amplified region was analyzed by quantitative PCR and percentage enrichment of each modification over input chromatin DNA was shown. ChIP PCR primers sequences are listed in the Supplementary Materials.

### Limiting dilution tumorigenic assay

To perform limiting dilutions (100, 500, 2500, 1 × 10^4^ and 1 × 10^5^) of ALDH^high^ cell sorted from stable Jarid2-sh 5637 and SCaBER cells, we mixed cells with Matrigel (BD biosciences) (1:1) and subcutaneously injected to nude mice. The animals injected with cancer cells were euthanized after 10 weeks.

### Statistics

All experiments were repeated at least three times, and the data were analyzed using the SPSS 12.0 statistical software package (SPSS, Inc.). Student's *t*-test was used to comparing the means of two samples. A value of p less than 0.05 (*P < 0.05) was regarded statistically significant. Data were presented as mean ± standard deviation.

## CONCLUSIONS

These results suggested that Jarid2 acted upon p16 to regulate stem-cell-property in TICs of bladder cancer. Collectively, the data indicate that Jarid2 is an essential regulator in bladder cancer cells and can be used as a novel therapeutic target in the treatment of the disease.

### Availability of data and materials

The raw data generated and analyzed during the current study are available from the corresponding author on reasonable request.

## SUPPLEMENTARY MATERIALS FIGURE



## References

[1] Knowles MA, Hurst CD (2015). Molecular biology of bladder cancer: new insights into pathogenesis and clinical diversity. Nat Rev Cancer.

[2] Chan KS, Volkmer JP, Weissman I (2010). Cancer stem cells in bladder cancer: a revisited and evolving concept. Curr Opin Urol.

[3] Falso MJ, Buchholz BA, White RW (2012). Stem-like cells in bladder cancer cell lines with differential sensitivity to cisplatin. Anticancer Res.

[4] Chan KS, Espinosa I, Chao M, Wong D, Ailles L, Diehn M, Gill H, Presti J, Chang HY, van de Rijn M, Shortliffe L, Weissman IL (2009). Identification, molecular characterization, clinical prognosis, and therapeutic targeting of human bladder tumor-initiating cells. Proc Natl Acad Sci USA.

[5] Jinesh GG, Choi W, Shah JB, Lee EK, Willis DL, Kamat AM (2013). Blebbishields, the emergency program for cancer stem cells: sphere formation and tumorigenesis after apoptosis. Cell Death Differ.

[6] Lin C, Song W, Bi X, Zhao J, Huang Z, Li Z, Zhou J, Cai J, Zhao H (2014). Recent advances in the ARID family: focusing on roles in human cancer. Onco Targets Ther.

[7] Pasini D, Cloos PA, Walfridsson J, Olsson L, Bukowski JP, Johansen JV, Bak M, Tommerup N, Rappsilber J, Helin K (2010). JARID2 regulates binding of the Polycomb repressive complex 2 to target genes in ES cells. Nature.

[8] Peng JC, Valouev A, Swigut T, Zhang J, Zhao Y, Sidow A, Wysocka J (2009). Jarid2/Jumonji coordinates control of PRC2 enzymatic activity and target gene occupancy in pluripotent cells. Cell.

[9] Shen X, Kim W, Fujiwara Y, Simon MD, Liu Y, Mysliwiec MR, Yuan GC, Lee Y, Orkin SH (2009). Jumonji modulates polycomb activity and self-renewal versus differentiation of stem cells. Cell.

[10] Tange S, Oktyabri D, Terashima M, Ishimura A, Suzuki T (2014). JARID2 is involved in transforming growth factor-beta-induced epithelial-mesenchymal transition of lung and colon cancer cell lines. PLoS One.

[11] Lei X, Xu JF, Chang RM, Fang F, Zuo CH, Yang LY (2016). JARID2 promotes invasion and metastasis of hepatocellular carcinoma by facilitating epithelial-mesenchymal transition through PTEN/AKT signaling. Oncotarget.

[12] Oguro H, Iwama A, Morita Y, Kamijo T, van Lohuizen M, Nakauchi H (2006). Differential impact of Ink4a and Arf on hematopoietic stem cells and their bone marrow microenvironment in Bmi1-deficient mice. J Exp Med.

[13] Bracken AP, Kleine-Kohlbrecher D, Dietrich N, Pasini D, Gargiulo G, Beekman C, Theilgaard-Monch K, Minucci S, Porse BT, Marine JC, Hansen KH, Helin K (2007). The Polycomb group proteins bind throughout the INK4A-ARF locus and are disassociated in senescent cells. Genes Dev.

[14] Landeira D, Sauer S, Poot R, Dvorkina M, Mazzarella L, Jorgensen HF, Pereira CF, Leleu M, Piccolo FM, Spivakov M, Brookes E, Pombo A, Fisher C (2010). Jarid2 is a PRC2 component in embryonic stem cells required for multi-lineage differentiation and recruitment of PRC1 and RNA Polymerase II to developmental regulators. Nat Cell Biol.

[15] Liang Y, Zhu F, Zhang H, Chen D, Zhang X, Gao Q, Li Y (2016). Conditional ablation of TGF-β signaling inhibits tumor progression and invasion in an induced mouse bladder cancer model. Scientific Reports.

[16] Li G, Margueron R, Ku M, Chambon P, Bernstein BE, Reinberg D (2010). Jarid2 and PRC2, partners in regulating gene expression. Genes Dev.

[17] Son J, Shen SS, Margueron R, Reinberg D (2013). Nucleosome-binding activities within JARID2 and EZH1 regulate the function of PRC2 on chromatin. Genes Dev.

[18] Arima Y, Hayashi N, Hayashi H, Sasaki M, Kai K, Sugihara E, Abe E, Yoshida A, Mikami S, Nakamura S, Saya H (2012). Loss of p16 expression is associated with the stem cell characteristics of surface markers and therapeutic resistance in estrogen receptor-negative breast cancer. Int J Cancer.

[19] Peng L, Hu Y, Chen D, Jiao S, Sun S (2016). Ubiquitin specific peptidase 21 regulates interleukin-8 expression, stem-cell like property of human renal cell carcinoma. Oncotarget.

[20] Chen D, Jarrell A, Guo C, Lang R, Atit R (2012). Dermal beta-catenin activity in response to epidermal Wnt ligands is required for fibroblast proliferation and hair follicle initiation. Development.

[21] Budnick I, Hamburg-Shields E, Chen D, Torre E, Jarrell A, Akhtar-Zaidi B, Cordovan O, Spitale RC, Scacheri P, Atit RP (2016). Defining the identity of mouse embryonic dermal fibroblasts. Genesis.

[22] Pei M, Chen D, Li J, Wei L (2009). Histone deacetylase 4 promotes TGF-beta1-induced synovium-derived stem cell chondrogenesis but inhibits chondrogenically differentiated stem cell hypertrophy. Differentiation.

