# Supplementary material for: Jarid2 is essential for the maintenance of tumor initiating cells in bladder cancer
Source: Oncotarget. 2017 Feb 20;8(15):24483–90. doi: 10.18632/oncotarget.15522 (PMC5421864; doi:10.18632/oncotarget.15522)
Supplement: Supplementary file 1 [file oncotarget-08-24483-s001.pdf]

Jarid2 is essential for the maintenance of tumor initiating cells in bladder cancer

Supplementary Materials

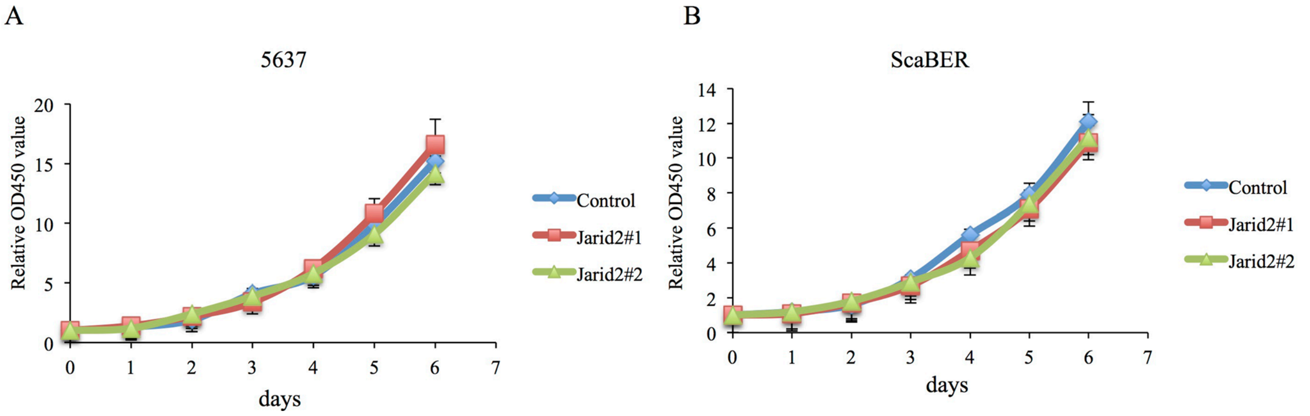

Supplementary Figure 1: Jarid2 does not affect bladder cancer cell proliferation. (**A**) Cell proliferation of 5637 cells transfected with control or Jarid2 siRNA at various time points were measured by MTT assays. (**B**) Cell proliferation of SCaBER cells transfected with control or Jarid2 siRNA at various time points were measured by MTT assays. Data represent the mean (± s.d.) of three independent experiments, each performed in triplicate.
